# Weight Gain During Treatment of Bipolar Disorder (BD)—Facts and Therapeutic Options

**DOI:** 10.3389/fnut.2019.00076

**Published:** 2019-06-11

**Authors:** Harald Mangge, Susanne Bengesser, Nina Dalkner, Armin Birner, Frederike Fellendorf, Martina Platzer, Robert Queissner, Rene Pilz, Alexander Maget, Bernd Reininghaus, Carlo Hamm, Konstantin Bauer, Alexandra Rieger, Sieglinde Zelzer, Dietmar Fuchs, Eva Reininghaus

**Affiliations:** ^1^Clinical Institute of Medical and Chemical Laboratory Diagnosis, Medical University of Graz, Graz, Austria; ^2^Department of Psychiatry and Psychotherapeutic Medicine, Medical University of Graz, Graz, Austria; ^3^Division of Biological Chemistry, Biocenter, Medical University of Innsbruck, Innsbruck, Austria

**Keywords:** bipolar disorder, weight gain, inflammation, obesity, oxidative stress, biochemistry

## Abstract

Bipolar disorder (BPD) is a mood disorder, which is characterized by alternating affective states, namely (hypo)mania, depression, and euthymia. Evidence is growing that BPD has indeed a biologic substrate characterized by chronic inflammation, oxidative stress, and disturbed energy metabolism. Apart from this, there is obviously a hereditary component of this disease with multi-genetic factors. Most probably a susceptibility threshold favors the outbreak of clinical disease after a cascade of stress events that remain to be elucidated in more detail. Evidence is also growing that weak points in brain energy metabolism contribute to outbreak and severity of BPD. Conventional psychopharmacologic therapy must be reassessed under the aspects of weight cycling and development of central obesity as a deterioration factor for a worse clinical course leading to early cardiovascular events in BPD subgroups.

## Introduction

Bipolar disorder (BD) is a mood disease, which is characterized by alternating affective states between the poles of euphoria or dysphoria, euthymia, and depression (1, 2). Symptoms of mania include over at least 7 days of euphoria or dysphoria or increased energy as major symptoms, and additional symptoms like inflated self-esteem or grandiosity, decreased need for sleep, logorrhea, flight of ideas, racing thoughts, distractibility, increased goal-directed activities, excessive involvement in pleasurable activities that have a high potential for painful consequences (e.g., like unrestrained buying sprees or foolish business investments). The latter symptoms can also occur as sub-manic form in episodes of hypomania not causing psycho-socio-economic damages. Depressive episodes are characterized by the major symptoms of depressed mood, anhedonia, loss of interests, and loss of energy as well as additional symptoms like changes of appetite, slowing down of thoughts and a reduction of physical movement, feelings of worthlessness or excessive or inappropriate guilt, loss of concentration or indecisiveness, and recurrent thoughts of death or recurrent suicidal ideation.

The course of illness is classified according to the current revision of the DSM 5—as BD I, BD II and cyclothymic disorder. Individuals with BD I suffer from recurrent episodes of manic, depressed, or mixed episodes. In contrast, individuals with BD II disorder present only hypomanic and depressed episodes. Individuals with cyclothymic disorder show over at least 2 years symptoms of dysthymic mood and mild hypomanic episodes, which do not fulfill the criteria for bipolar II disorder or major depressive disorder (MDD) (1).

**Figure 1:**
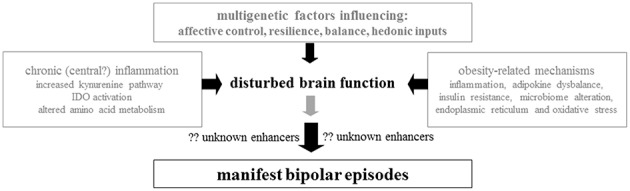
The biologic trace of bipolar disorder: Involvement of genetic predisposition, inflammation, amino acid metabolism, endoplasmic reticulum stress, obesity/adipokines, nutrition/microbiome, and oxidative stress.

According to current scientific knowledge, the occurrence of BD is best explained by the vulnerability-stress-model, which links a strong genetic heritability, chronic stress, and acute triggers (2). An orchestra of risk genes leads to alterations in multiple pathways like neurotransmitter systems (e.g., dopaminergic, serotonergic, and glutamatergic pathways), neurodevelopment, synaptogenesis, neurotransmission, and circadian rhythms (2). The polygenic predisposition and gene-environment interactions lead to the outbreak of the mood disorder. Epigenetic changes of gene expression regulation can mitigate the gene-environment interactions. Nutritional factors, as well as microbiome diversity affect the human body on gene expression and epigenetic levels (3). Taken together, multiple disease mechanisms like neurotransmitter imbalances, disturbed circadian rhythms, changes in neurodevelopment and neuroplasticity, endoplasmic reticulum (ER) stress and oxidative stress, chronic inflammation, and immunological reactions are propagating factors in BD (4–11). Cellular stressors especially, which are commonly caused by obesity and chronic inflammation, have been in the spotlight of research for many years (12, 13) (Figure 1).

### Weight Gain as Psychopharmacological Side Effect

Individuals with BD frequently suffer from uncontrolled weight gain and cardiovascular disease (CVD) (12, 13). Accordingly, by diverse metabolomics studies of cerebrospinal fluid, post-mortem brain tissue and blood, BD was associated with changes in energy metabolism (14, 15).

Moreover, BD was particularly associated with mitochondrial dysfunction in serum metabolomics analyses. The citric acid cycle, urea cycle, and amino acid metabolism were each affected in BD and may, to some degree, explain dysregulation in obesity related pathways (15). Nevertheless, it is still unclear if BD is causative in this context or if the psychopharmacologic therapy of BD induces uncontrolled weight gain and consecutively CVD.

It is well-known that some kinds of psychopharmacological medication, basically sedating antidepressants (e.g., mirtazapine, amitriptyline, clomipramine) and sedating atypical antipsychotics (e.g., olanzapine, quetiapine), as well as mood stabilizing agents (e.g., lithium and the anticonvulsant valproate) can lead to increased appetite and associated weight gain (16). Yet, not all individuals treated with mood stabilizing agents or antidepressants gain weight equally, and medicament-naive individuals with BD also suffer from weight gain.

An individual's genetic predisposition seems to influence the association between BD and obesity additionally. The phenotype of antipsychotics induced weight gain shows polygenic inheritance. For example, one genome wide association study (GWAS), which analyzed antipsychotic-induced weight gain (AIWG) found a genome wide association of AIWG with gene variants in the *PTPRD* gene (*protein tyrosine phosphatase, receptor type D*, 9p24–p23; rs10977144), and *GFPT2* gene (*glutamine-fructose-6-phosphate aminotransferase 2*, 5q35.3; rs12386481) (17). Nevertheless, an orchestra of gene variants (e.g., *HTR2C, leptin genes, DRD2, TNF, SNAP-25, MC4R, CNR1, MDR1, ADRA1A, INSIG2*) is associated with AIWG and comprehensive reviews are summarizing this topic (18).

Valproate and lithium also feature weight gain as a side effect and both interact with HDACs (histone deacetylases), which are important enzymes for epigenetic modulation by catalyzing the deacetylation of histones. The deacetylation of histones induces a tighter wrapping of proteins around the DNA and blocks transcription by physically limiting the access of transcription factors (19, 20). Valproate also induces the recruitment of methyl cytosine binding protein 2 (MeCP2), which directs the way of HDACs to the regulatory site, leading to repression of transcription by deacetylation of the histones and chromatin condensation around methylated DNA. Thus, MeCP2 acts as transcription inhibitors. Taken together, valproate affects local DNA methylation, deacetylation, and demethylation of activated histones and recruitment of inhibitory complexes (21). There are no studies available concerning valproate associated weight gain and associated epigenetic changes, but since valproate interferes with the recruitment of MeCP2 there may be a relationship between epigenetic changes and valproate induced obesity.

Literature shows for example that patients suffering from mild Rett syndrome, which includes mutations in MeCP2, commonly show obesity (22). Furthermore, MeCP2 knockout mice show hyperphagia, increased high fat diet craving and obesity (23, 24). MeCP2 mRNA levels were also upregulated in patients with bipolar II disorder (25), which shows again that obesity related pathways are on one hand altered in medication associated obesity, but on the other hand in BD itself.

### Obesity as a Trait Marker for BD Itself

Even though psychopharmacological side effects can to some degree explain weight gain and obesity in BD, the association still exists even when controlled for psychopharmacological medication in statistical analyses. There are globally diverse research groups, which analyse obesity as a trait marker for BD itself, as the observed increased rate of obesity cannot be explained by medication alone (12, 13). To further investigate shared pathways between obesity and BD the “BIPFAT study” has been established by E.R. at the Medical University of Graz (6, 11, 26–30). In a series of publications, a biomarker profile was established, which indicates a combination of inflammatory and oxidative features and subtle abnormalities of the energy metabolism (Figure 1). Herein, we review these results and discuss the clinical relevance of the new data against the backdrop of pre-existing literature.

### Adipokines, Inflammation and Amino Acid Metabolism

Adipokine alterations were associated with BD in previous literature (31). Platzer et al. found significantly lower adiponectin levels in fasting blood of female depressive patients with BD compared to healthy controls (32).

Adiponectin, a collagen-like adipocytokine is exclusively and abundantly expressed in adipose tissue. It circulates in peripheral blood in low molecular weight (LMW), medium molecular weight (MMW), and high molecular weight (HMW) isoforms also called sub fractions or multimers (33). It has been reported that total adiponectin is decreased in obese persons with cardiovascular risk factors, such as diabetes and dyslipidemia. Evidence exists that adiponectin exerts a protective effect against atherosclerosis, due to profound anti-inflammatory and anti-atherogenic features (34–36). These protective vascular functions of adiponectin are mainly mediated by the HMW subfraction (33). Thus, not only are total adiponectin levels critical for pathogenic effects, but also the percentages of the three isoforms within the total molecule fraction. We provided first evidence that HMW adiponectin is involved in early atherosclerosis of obese juveniles (33). Increased LMW/total adiponectin ratios are associated with a critical subcutaneous adipose tissue (SAT) topography (i.e., extended nuchal fat thickness), which is linked to increased cardiovascular risk (37) and premature genomic aging (38).

Since adiponectin exerts anti-inflammatory properties, the reduction of total adiponectin in BD may explain to some degree the proneness toward a mild chronic inflammation seen in BD (39). This could be one link between mood disorders, obesity, and metabolic disturbances, as well as inflammation. Thus, the question arises of which isoforms of adiponectin are more or less involved in BD. To the best of our knowledge, we did not find any study investigating adiponectin isoforms in addition to total adiponectin levels in BD. Unfortunately; we did not yet analyze HMW, LMW, and LMW adiponectin in our BD sample. Nevertheless, it will be interesting to analyze this in future in our BIPFAT cohort. It is like that this analysis will be the key to a better understanding of the role of adiponectin in BD because in both anorexia nervosa (40) and patients with type 1 and type 2 diabetes (41) a decreased HMW/total adiponectin and an increased LMW/total adiponectin ratio is associated with increased depressive/psychiatric symptoms.

In another investigation a positive correlation between high sensitive (hs) C-reactive protein and the number of manic and depressive episodes was shown in females with BD (42). The mild inflammation also leads to activation of the enzyme IDO, which degrades the amino acid tryptophan. Thus, overweight/obese individuals with BD also have an increased metabolism of tryptophan down to the kynurenine (KYN) pathways, indicated by an increased serum KYN-to-tryptophan ratio (30, 43). This observation may also indicate an increased immune-inflammatory activity and stress in BD (43).

### Oxidative Stress and Endoplasmic Reticulum (ER) Stress

Oxidative stress and ER stress have been associated with BD in diverse lines of evidence (44). Oxidative stress, which results from an imbalance between reactive oxygen species (ROS) and the antioxidative defense, was associated with oxidative stress in hitherto literature. The oxidative stress seems to fluctuate depending on affective episodes and energy level. In manic episodes some authors even compare the amount of oxidative stress with conditions found in septic patients. There seems to be a concatenation between peripheral markers of oxidative stress such as malondialdehyde (MDA) and carbonyl proteins, antioxidative parameters superoxide dismutase (SOD), glutathione S-transferase (GST) and total antioxidative capacity (TAC), and disease mechanisms of BD (4, 10, 45–51). In the “BIPFAT study” we found significantly reduced TAC and lipid oxidation [Malondialdehyde (MDA)] in fasting blood of euthymic patients with BD compared to controls. Furthermore, small sex and anthropometrics effects on peripheral oxidative stress markers were found (28). Summarized, patients with BD show a misbalance in oxidative stress pathways and the antioxidative defense, in terms of continuous chronic, mild oxidative stress with excessive consumption of antioxidants which results in significantly decreased TAC. Lithium taking participants had the lowest serum MDA levels (analyzed by gas chromatograph mass spectrometry) (52) while atypical antipsychotics (AAP) taking persons had higher oxidative stress markers. Hence, lithium shows antioxidative effects in addition to the well-known mood stabilizing action (27).

Oxidative stress and ER stress have a reciprocal relationship. ER stress and changes in the related unfolded protein response (UPR) can induce ROS. The other way oxidative stress can affect the correct folding of proteins, which can induce ER stress is by misfolding or unfolding of proteins (53, 54). Obesity, calcium depletion, viral infections, mutations leading to misfolding of proteins, hypoxia, energy depletion lead as well to misfolding/unfolding of proteins and toxic ER stress (55, 56). ER stress and its rescue pathways, the UPR, were associated with BD in various *in-vitro* studies (57–59). The peripheral blood markers of ER stress were significantly altered in BD. *BiP* gene expression was significantly increased and unspliced *XBP1* (but not the *XBP1* splicing event itself) was significantly decreased in fasting blood of patients with BD compared to healthy controls in the “BIPFAT study” (9).

### Nutrition and Microbiome

Oxidative stress often results from an imbalance between ROS and antioxidative agents. Antioxidants from a healthy, well-balanced and vitamin rich diet have positive effect on mood, which is propagated by the popular field of “nutritional psychiatry” (60). In this context, the gut-brain-axis, which is a bidirectional communication system between the gut-microbiota and the brain, has been a large field of scientific investigations in recent years. The gut-brain axis interferes with the human organism on metabolomics-, transcriptomics- and transmitter levels as the gut-microbiota affect tryptophan availability and tryptophan metabolism, as well as serotonin synthesis. Furthermore, the gut-microbiota influence the vagal tone via released transmitters. Gut-microbiota can also affect gene expression and epigenetic transcription regulation by processed nutrients (e.g., polyphenols from coffee, green tea, or aronia juice) or by short chain fatty acids like butyrate and proprionate (61). The gut microbiome diversity also correlated negatively with the methylation of the clock gene *ARNTL* in fasting blood DNA of BD study participants (3). Mechanisms of the gut-brain axis have been discussed as modifying factors in BD disease mechanisms (61–64). Microbial alpha-diversity even correlated negatively with illness duration in BD, which underlines the importance of healthy nutrition and a balanced gut-microbiome for mood regulation (61). The gut-microbiota diversity associates with diverse environmental factors like an unbalanced diet, lack of physical activity, inflammation, and oxidative stress (65–67). In this context it is interesting that individuals with BD showed a significantly reduced TAC in our study, which can either be explained by a chronic compensation of oxidative stress (and consequently an excessive consumption of antioxidative acting mediators) based on chronic oxidative stress in BD but also be caused by a misbalance of vitamin intake based on a poor diet of patients with BD (28). Unhealthy diet patterns may be based on increased food craving, which may be a compensation mechanism of depressed patients to encounter tryptophan depletion and serotonin deficit. Dalkner et al. (43) investigated food craving in BD compared to healthy controls and found a positive correlation between high carbohydrate craving and kynurenine (KYN) serum concentrations, as well as the KYN-to-tryptophan ratio. Additionally, overweight or obese individuals with BD showed increased craving for tryptophan. Dalkner et al. also observed increased fat craving in males vs. females with BD (43). In line with this, Fellendorf et al. found a decrease of the branched chain amino acid leucine in BD compared to controls and associations between leucine, valine, isoleucine, and anthropometric as well as glucose metabolism in the BD cohort (68).

### The Influence of Obesity on Course of BD and Cognitive Function

Adults with BD with excess weight are not only more susceptible to a relapse-prone course of illness, but are also more likely to suffer from weight cycling. Patients with obesity and weight cycling also show a worse course of disease and increased predisposition for CVD (26). The finding of elevated pro-inflammatory cytokines in this cohort may identify a separate subpopulation with greater susceptibility to CVD. Relating to the overarching aim of personalized treatment and preventive strategies in BD, our results provide preliminary support for stratifying BD cardiovascular risk based on anthropometrics and weight cycling (26). In this context, individuals with BD in general show an increased central body fat accumulation i.e., higher subcutaneous adipose tissue at upper abdomen, accompanied by metabolic syndrome (69). Obesity and metabolic syndrome go in line with a worse course of disease as well as impaired cognitive function even in euthymia (70). Interestingly, male euthymic BD patients had a significant negative correlation between the performance in the California verbal learning test (CVLT) and the 3-hydroxykynurenine to kynurenic acid ratio (71). This suggests that a shift toward the hydroxykynurenine arm of the KYN pathway may be associated with poorer memory performance due to its effects on neuronal functioning and neurogenesis in the hippocampus. Central inflammatory processes in individuals with BD may be of high importance in these altered cognitive functions by favoring this metabolic pathway (71). Moreover, it was found that female individuals with BD who performed vigorous physical activity performed significantly better in cognitive tasks, especially in memory tasks, compared to individuals with low and moderate physical activity (72).

### Chronic Inflammation and KYN Pathways

Tryptophan has been in the center of psychiatric research for decades, because it is an essential amino acid and a precursor of serotonin (5-hydroxytryptamine/5HT). There is also a second critical biochemical pathway which involves tryptophan, namely the formation of KYN (11). The enzyme Indoleamine 2,3-dioxygenase-1 (IDO) converts tryptophan into KYN, which leads to reduced tryptophan availability for 5HT synthesis. However, as central nervous regulation systems may be able to compensate for the inflammation-induced decrease in circulating tryptophan, the serotonergic system may not be affected unfavorably as part of this process. Therefore, the KYN-pathway might be one of the mechanisms associated with the development of affective symptoms (11, 30). Thus, the mood disorder BD has been linked with changes in immune-inflammatory pathways and tryptophan catabolites (TRYCATs) (11, 30). The activation of immune-inflammatory biochemical mechanisms, e.g., pro-inflammatory cytokine levels that are associated with changes in the TRYCAT metabolism, was in turn associated with manic and depressive episodes in BD (11, 30). Taken together, increased tryptophan breakdown and higher levels of the immune system biomarker neopterin in overweight/obese individuals with BD compared to normal-weight patients, indirectly underline the presence of immune-mediated inflammation. Chronic low-grade inflammation contributes to the high prevalence of CVD and increased mortality (73, 74). Increased neopterin, KYN levels, and an increased KYN/TRP ratio suggest an increased activity of tryptophan-degrading IDO in overweight/obese euthymic individuals with BD (30). Importantly, an increased KYN/TRP ratio was associated with a higher likelihood of fatal cardiovascular events in individuals without mental disorder (73, 74). Indirect mediators of immune-mediated inflammation (increased tryptophan breakdown and altered neopterin levels) suggest chronic inflammation as a link between BD and CVD (30).

### Obesity and Circadian Rhythms

Neurotransmitter levels are not only influenced by tryptophan breakdown, but also by enzymatic degradation (e.g., monoamine oxidase A [*MAOA*] and catechol-O-methyltransferase) (75). It is well-known, that the *MAOA* inhibitors have a robust antidepressant effect, which explains the central role of the *MAOA* candidate gene in early gene-association studies (76, 77). Interestingly, the MAOA gene transcription is activated by major players of the molecular 24 h clock, namely by transcription factors encoded by the clock genes *ARNTL* and *NPAS2* (78, 79). Thus, disturbed circadian rhythms are strongly interconnected with mood changes and it is not surprising that mood stabilizing agents like lithium and valproate interfere with the molecular circadian clock. Lithium and valproate inhibit *GSK3Beta*, which is responsible for the phosphorylation of the cycle length determining *PER3* gene products (19, 80, 81). In *in-vitro* lithium treatment leads also to rapid proteasomal degradation of the clock gene *REV-ERB-*α and activation of the clock gene *ARNTL* (82). Disturbed circadian rhythms are also associated with obesity, the metabolic syndrome, and cardiovascular risk, which underlines again the concatenation between the energy metabolism and mood swings. Obesity is also correlated with the epigenetic modification of core clock genes, which points to the reciprocal interaction between disturbed circadian rhythms, mood swings and obesity (83). Against this background there are multiple interactions between obesity and mood, which makes it impossible to solve the hen/egg problem!

### The Effect of Weight Management on Therapeutic Success in BD

The question to what extent weight gain or loss influences the therapeutic response in BD is still unanswered. Indirect evidence for a positive effect of weight loss on mood comes from obese patients showing an improved long-term depression outcome after bariatric surgery (84). In a smaller scale reduction, long-term improvements in anxiety severity scores have been observed (84). Nevertheless, more work is required to investigate the mechanistic and causal relationship between obesity and psychiatric disorders including BD (84). As stated before, patients with BD are at a substantially increased risk of obesity when compared to those without BD. Patients with obesity and BD have a greater illness severity and a poorer treatment outcome.

Chronic inflammation, decreased insulin action, and oxidative stress most likely address biologic weak points of BD patients and thus aggravate the clinical course of disease. It is possible that latent defects of the energy metabolism may represent one important weak point. Apart from this, increased social exclusion caused by obesity will open the way to psychiatric decompensation. An improved metabolic/inflammatory state and a stronger self-esteem after weight loss will pave the way for amelioration of disease drivers.

On the other hand, medications for BD associate with weight gain, highlighting the need for the development of more weight-neutral, effective treatments. Preventative interventions have shown some promise for preventing medication related weight gain and deserve further research attention. While there is some evidence for the use of behavioral and medical strategies for weight loss in BD, bariatric surgery may potentially be the most robust intervention in adipose patients with stable BD. While existing research supports the use of bariatric surgery in patients with a well-managed bipolar disorder, further longitudinal investigation is needed to address specific predictors of success in this population (85). On the other hand, a bigger part of problem is the unhealthy lifestyle in individuals with BD also leading to increased prevalence of obesity and metabolic syndrome. Different lifestyle interventions on psychotherapeutic, psychological or physical exercise levels have been effective (86–88). Interventions of ≥12-months duration compared to ≤ 6-months duration achieved more consistent outcomes, though effect sizes are similar for both shorter and longer duration interventions (87).

## Conclusions

Evidence is growing that BD has indeed a biologic basis characterized by chronic inflammation, oxidative stress, and disturbed energy metabolism (Figure 1). Apart from this, there is obviously a hereditary component of this disease with multi-genetic factors (89). Most probably a susceptibility threshold favors the outbreak of clinical disease after a cascade of stress events that remain to be elucidated in more detail. Evidence is also growing that weak points in brain energy metabolism contribute to outbreak and severity of BD. Conventional psychopharmacologic therapy must be reassessed under the aspects of weight cycling and development of central obesity as a deterioration factor for a worse clinical course leading to early cardiovascular events in BD subgroups.

## Author Contributions

All authors listed have made a substantial, direct and intellectual contribution to the work, and approved it for publication.

### Conflict of Interest Statement

The authors declare that the research was conducted in the absence of any commercial or financial relationships that could be construed as a potential conflict of interest.
